# No research for the decisionally-impaired mentally ill: a view from Montenegro

**DOI:** 10.1186/s12910-020-00489-z

**Published:** 2020-06-09

**Authors:** Tea Dakić

**Affiliations:** Clinical Center of Montenegro, Clinic for Psychiatry, Ljubljanska bb, Podgorica, 81000 Montenegro

**Keywords:** Psychiatry, Research, Policy, Inclusion, Consent, Mental disorders

## Abstract

**Background:**

Many of the important elements of a valid informed consent – comprehension, voluntariness, and capacity – can be compromised or unmet in the context of psychiatric research. The inability to protect their own interests puts mentally ill subjects at an increased likelihood of being wronged or harmed and makes them particularly vulnerable in the context of clinical research. Therefore, they are due extra protection. Sometimes, these additional safeguards can significantly limit the possibilities for research involving subjects deemed unable to consent due to their mental illness. Montenegro, a middle-income country in Southern-Eastern Europe, goes so far in their policy to protect these subjects from harms of research, as to ban all biomedical research on mentally ill persons who are unable to provide consent.

**Main body:**

Mental health research is often neglected and very low on the list of health research priorities, especially in low- and middle-income countries. Despite the fact that mental health disorders are among leading causes of disability, the need for evidence-based services and interventions for those affected remains unmet.

To exclude all members of a certain group of subjects seems extremely restrictive and unnecessary. Such a policy is discriminatory and unethical, because it inflicts further harms and exclusion of those patients from participation in society. This unjust exclusion policy obstructs research of certain psychiatric disorders and implies that new treatments for conditions that directly affect these incapacitated subjects will not be developed.

**Conclusions:**

Scientific and clinical development must not be precluded by overly restrictive, discriminatory and unjust practices, such as the normative ban on research on decisionally-impaired mentally ill subjects. Rather, there should be a regulative framework that ensures that those who cannot consent for themselves are respected and protected in research, the anticipated benefits maximized, risks minimized, their autonomy recognized and extended. These patient-subjects must be appropriately included unless there is a clear and compelling rationale and justification that inclusion is inappropriate with respect to the health of the participants or the purpose of the research.

## Background

Many of the important elements of a valid informed consent – comprehension, voluntariness, and capacity – can be compromised or unmet in the context of psychiatric research. Due to the inability to protect their own interests mentally ill patients face an increased likelihood of being wronged or harmed, which makes them particularly vulnerable in the context of clinical research. Therefore, they are due additional protection and safeguards [1].

Certain nations, such as Montenegro, however, go so far to protect these vulnerable subjects from burdens of research that they are completely excluding them from research opportunities. Namely, in 2013 the Montenegrin government passed the Law on Protection and Exercise of the Rights of the Mentally Ill, which explicitly proscribes research on persons who cannot provide informed consent due to their mental illness [2]. The background to this legal restriction remains unclear at this time. It is, however, clear that such a policy is discriminatory and unjust, as it deprives mentally ill of the direct and indirect benefits arising from participation in research. The best way to protect individuals from exploitation should not be to exclude them from participation in research, but to ensure the ethical design and scientific soundness of research, so that the rights and welfare of participants are respected while still generating valuable general knowledge.

## Main body

The first research ethics principle of the Nuremberg Code requires the informed and voluntary consent from the participant as a necessary precondition for research, regardless of the participant’s specific attributes. Particularly, the Code declares *that the person involved should have legal capacity to give consent … and should have sufficient knowledge and comprehension of the elements of the subject matter involved as to enable him to make an understanding and enlightened decision* [3], p.181.

This postulate seems to be justified by the intention to address highly risky non-therapeutic research on the easily coerced populations [4]. Accordingly, this formulation completely excludes those who lack the legal, mental or physical capacity to consent from research [4]. On the other hand, the Nuremberg Code also states that research on human subjects is aimed at producing benefits for the society that are “unprocurable by other methods or means of study” [3]. Therefore, such an extensively exclusive policy, as specified through the first principle of the Nuremberg Code, would bring about undesirable consequences for society. For instance, it would preclude obtaining important knowledge about those conditions that result in vulnerability or loss of competence [5]. This concern was positively recognized and it is presently reflected through internationally established research ethics framework. Well-known contemporary codes and regulations now do allow for research on persons unable to provide consent, as long as the research is ethically and scientifically justified and additional safeguards are in place for these participants [6–8].

It is now widely agreed that research on a person whose capacity to consent is compromised may be undertaken if it is methodologically necessary to use such participants, and if the research is likely either to benefit the participants themselves or to benefit others with the same capacity-limiting condition. The research should, however, impose no more than minimal risk to the participants. Further conditions frequently required for safeguarding interests of the participants concerned are that consent be acquired from a legally authorized representative (LAR) of the participant, that assent should be sought from the participant themselves, and their dissent be respected [9].

The 2013 revision of the Declaration of Helsinki, as developed by The World Medical Association reaffirms these agreed-upon recommendations for research subjects who are incapable of giving informed consent, and proposes an additional requirement that research involving these subjects may be done only if the physical or mental condition that prevents giving informed consent is a necessary characteristic of the research population [6].

Similarly, according to the *Additional Protocol to the European Convention on Human Rights and Biomedicine, concerning Biomedical Research* [7], to which Montenegro is a signatory, research on a person whose capacity to consent is compromised may be undertaken if there is a likely benefit for them and if the research cannot be performed with persons capable of providing informed consent. Additionally, this protocol allows for research that does not have the potential to directly benefit the participant, if it is intended to promote the health of the group the participant belongs to and if it entails only minimal risks and minimal burdens. These conditions significantly limit the possibilities for research involving participants unable to consent due to their mental illness, but do allow for the research necessary to understand and develop new treatments for these conditions.

The 2005 version of the Montenegrin Law on Protection and Exercise of the Rights of the Mentally Ill provided for the safeguards for mentally ill who are unable to consent to research identical to those of the *Additional Protocol to the European Convention on Human Rights and Biomedicine, concerning Biomedical Research* [7, 10]. The other notable healthcare laws in Montenegro, the Law on Medicines and the Law on Patients’ Rights, both adopted in 2011, also allow for research on individuals who lack capacity to consent to it under similar conditions [11, 12]. Under these two laws, minors and other individuals who are incapable of providing their consent may be included in therapeutic research if the authorization for being subjected to research is given by the parent or other legal representative, and if the research of comparable effectiveness cannot be carried out on other less vulnerable individuals [11, 12].

Yet, in 2013 the Montenegrin government decided to single out mental disorders as the underlying cause of decision-making inability and introduce a particular piece of legislation that denies persons suffering from an incapacitating mental illness the opportunity to participate in research [2]. The most recent revision of the Law on Protection and Exercise of the Rights of the Mentally Ill, adopted in 2013, introduced “additional protections” for those vulnerable individuals who lack the capacity to provide consent due to their mental illness, by precluding any research on them [2]. This revision to Montenegrin law now only allows research on mentally ill who can consent for themselves, and bans biomedical research on mentally ill persons who are unable to give their consent.

The background for this normative change in legislation remains unknown. There are, however, speculations in scientific circles that it may relate to the fact that it was in 2013 that Montenegro was deemed one of the poorest states with high level of corruption as measured by Transparency International [13]. Furthermore, Montenegro was concurrently included in the Euro Health Consumer Index (EHCI), a public measurement of how national healthcare systems perform. That same year, Montenegro scored second worse (34th out of 35 countries) at EHCI, as a country with a poorly performing low quality health care system [14]. The modification of mental health legislation may have been intended to protect the rights of these very vulnerable patients in the setting of a poorly graded and corrupted healthcare system, which is likely prone to undue monetary inducements. More often than not, persons who suffer from severe mental disorders are socio-economically deprived. Offering payment to these prospective research participants who are unable to consent for themselves may further compromise the voluntariness of their assent or unduly influence the consent of their legal representatives. The change in legislation may have been reflective of the worry that only by determining the ethical and legal unacceptability of research on the mentally incapacitated participants can they be protected from undue inducements, coercion and exploitation.

However well-meant this change in legislation may be, excluding all members of a certain group of subjects is extremely restrictive and unnecessary. Such a practice perpetuates exclusion of those patients from participation in society and discriminates against them as potential research participants based on their undeserved characteristics [15].

### Mental disorders and decision-making ability

According to the World Health Organization (WHO), mental disorders represent a set of clinically recognizable symptoms characterized by a combination of abnormal thoughts, perceptions, emotions, behavior and relationships with others [16]. Although some individuals suffering from mental disorders may well be capable of making autonomous decisions, the very definition of these conditions offers the probability of compromised decision-making. Severely demented, intellectually impaired, or actively psychotic persons would, in general, be considered incapable of making autonomous decisions regarding their health care or research participation. Besides, there are other conditions which affect logical thinking and understanding of relevant information. These include severe anxiety, severe depression, mania, and delirium [17]. Because of their altered mental status persons with these conditions may lack the capacity to sufficiently understand information and make informed choices which makes them vulnerable to abuse [18].

Group of adults with mental illness that may render them unable to consent to research include: 1) those with intellectual disabilities who were never fully competent; 2) psychiatric patients with fluctuating capacity (i.e. they may have capacity when their illness is in remission but are incapacitated when their illness is more severe or untreated); and 3) individuals who suffer from dementia or other neurodegenerative diseases who once had but have since lost (and may never again regain) capacity [18]. These potential participants frequently lack the capacity to sufficiently understand information relevant to their decision to participate in research, and are thus granted special protections from being unjustly exploited.

While these mentally-disabling conditions may diminish a person’s decision making capacity, they certainly must not diminish their human rights. The United Nations Convention on the Rights of Persons with Disabilities sets out specifically that disabled individuals have the right to equal recognition before the law, the right to enjoy legal capacity and the right to exercise that capacity [19]. Nevertheless, the Civil Procedures Act of Montenegro, as adopted in 2015, still offers the possibility for an adult to be fully or partially deprived of their legal capacity based on disability [20]. The Civil Procedures Act is yet another piece of legislation that reflects an out-dated imperative to protect people with reduced decision-making capacity that does not recognize their ability to make decisions with support [21]. In this substituted decision-making model, as offered through the Civil Act, the person who is deprived of their legal capacity is appointed a guardian who makes decisions on behalf of the person with mental disability, while this person retains no power to make decisions and no say in matters concerning them, including health care and research. Article 12 of the United Nations Convention on the Rights of Persons with Disabilities states that persons with mental disabilities must not be denied the right to make medical decisions on the basis of impaired decision-making capacity and advocates for replacement of substitute decision-making by supported decision-making. Still, provisions embedded in Montenegrin legislation reflect yet another stigma regarding cognitive impairment due to mental disorder and a view that persons with severe mental disorders as not capable of making decisions and only needing care and protection [21]. These overly-protective measures rely on a solution that prevents persons with mental disabilities from making decisions, because they are considered “incompetent” and legally seen as “incapable” of doing so [22].

In case of Montenegro, where individuals with cognitive impairment usually have their legal capacity taken away or restricted based on their disability, mental capacity becomes synonymous with legal capacity, despite recommendations set out under the United Nations Convention on the Rights of Persons with Disabilities [22]. Without legal capacity, a person lacks agency as a rights holder and lacks the ability to make decisions in many aspects of life, including the ability to decide on their participation in research and the ability to provide consent [23]. In a country where mentally disabled persons are often stripped of the legal right to choose on an equal basis with others, the prohibition of research is yet another burden and another denial of the ability to be recognized as a rights holder before the law. Thus, the legal ban on research is more substantial and far-ranging than it would be should the assessment of the ability to consent be functional and not status or category based. Taking this into account, it is safe to assume that a considerable number of patients with cognitive impairments and limited mental and legal capacity will be adversely affected by the piece of legislation that paradoxically relates to the protection and exercise of their rights. Lack of mental capacity, as put under this law, presents yet another persistent barrier for these disabled individuals to exercise their rights. Namely the right to equality of opportunity and fair access to research directly, in terms of participation in research as well as indirectly, in terms of access to the results of research [24].

### The need for research

According to the World Health Organization (WHO), the burden of mental disorders continues to grow and substantially affects not only health, but also the economy and the human rights situation all around the world [25]. Despite the fact that mental health disorders are among top three most important contributors to global years lived with disability [26], the need for evidence-based services and interventions remains unmet. Mental health research is often neglected and very low on the list of health research priorities, especially in low and middle income countries (LMIC) [27]. The treatment gap for persons suffering from mental disorders exceeds 50% globally and is even larger in LMIC [28]. Providing safe and effective treatments for these patients and bridging a huge treatment gap in these countries is dependent on the condition that mentally ill individuals are given the opportunity to participate in research. Research is necessary in order to understand this gap, provide information on quantity and quality of unmet needs, offer socially valuable knowledge, build mental health care capacities accordingly and deliver adequate evidence-based interventions and services, particularly in LIMC countries like Montenegro [29].

### Implications and the results of the policy of exclusion from research

The exclusion of those who lack the capacity to give informed consent from research may be perceived as well intended. This approach could be based on the cautious idea that it is better to protect the participants from undue risks even if that means that they are largely excluded from research. However, this systematic exclusion does not come without a price. An additional cost and indirect harm of this strict protectionist model is that it slows down and prevents medical advances and new clinical approaches to treat the diseases from which these vulnerable individuals suffer, the very conditions that are often the underlying cause of their incapacity.

Special measures that are meant to protect the rights of those with mental illness paradoxically impede advances in treatment and care for these patients. To disallow enrollment in research of persons who lack the capacity to consent also means to prevent those individuals from receiving the potential benefits that research might offer them. For example, they may directly benefit clinically as a result of participating in research designed to develop new treatments for their condition. Even if there is no direct clinical benefit, these participants might benefit indirectly, as the research provides increased knowledge and understanding of these mental disorders and may eventually lead to better treatment and prevention [30]. Unless there is a valid scientifically grounded reason for it, excluding all members of a potentially vulnerable group of participants from research seems excessively restrictive. Moreover, such a practice is discriminatory, because it marginalizes all members of the group and prevents them from fully participating in society [15].

In accordance with the fundamental human freedoms and rights set out in the UN Principles for the Protection of Persons with Mental Illness and the Improvement of Mental Health Care [31], every patient has the right to receive health and social care and treatment as is appropriate to their health needs and in accordance with the same standards applicable to other ill persons. Practical realization of this principle is dependent on enabling health-related research on decisionally-impaired persons with mental disorders, under the same conditions that apply to all individuals who are unable to consent for themselves.

The UN Principles for the Protection of Persons with Mental Illness and the Improvement of Mental Health Care further declare [31]:

*There shall be no discrimination on the grounds of mental illness. “Discrimination” means any distinction, exclusion or preference that has the effect of nullifying or impairing equal enjoyment of rights. Special measures solely to protect the rights, or secure the advancement, of persons with mental illness shall not be deemed to be discriminatory.*

While the 2013 Montenegrin Law on Protection and Exercise of the Rights of the Mentally Ill may be protecting the most vulnerable individuals from the burdens of research and potential exploitation, it is still impairing their equal enjoyment of the right to benefits arising from participation in research. Special measures, as laid out in this law, fail to ensure their fair participation in society and stand in the way of efforts to ensure advancement in developing new treatments and preventive measures. Thus, the deliberate exclusion from research of persons with mental illness that deems them incapacitated is clearly discriminatory.

Safe and effective treatment, prevention, or diagnostics cannot be achieved without evidence. The social and scientific value of research is reflected in generating relevant evidence and generalizable knowledge, which are the resources necessary for protection and promotion of people’s health. The information obtained through research bears direct relevance for understanding or intervening on a significant health problem [8].

Just like other individuals and groups whose circumstances may make them vulnerable in the context of research, individuals who are incapacitated due to their mental illness should neither be inappropriately included nor automatically excluded from participation in research on the basis of their vulnerability. While present-day international recommendations and regulations on research on potentially vulnerable groups and individuals are moving towards requiring their appropriate inclusion in research so that the health needs of these groups may be adequately met [6–8], Montenegro is moving backward. The 2103 revision of the Montenegrin Law on Protection and Exercise of the Rights of the Mentally Ill that brought about a complete ban on biomedical research on mentally ill individuals who are unable to provide informed consent [2], is no step forward from the Nuremberg Code [3]. Montenegro, a developing middle income country in Southern-Eastern Europe, has a fairly young and developing system of ethical review of research on human subjects. It is perhaps not surprising that the decision-makers reached out to the very beginnings of research ethics and the milestone document that set the ground for protection of vulnerable subjects against unconsented research activities. Whatever prompted the change to a more restrictive Law on Protection and Exercise of the Rights of the Mentally Ill, the decision to exclude a group of decisionally-impaired mentally ill participants from research represents a major obstruction to realization of their right to be recognized and treated before law as persons with the same rights as others.

Therefore, I offer a set of recommendations for research with persons who are unable to provide consent for themselves due to their mental illness, so that they may be appropriately included in valuable research, while their dignity and rights are protected and their autonomy extended and respected.

### Set of recommendations for research with persons who are unable to provide consent due to their mental illness

Ever since Montenegro begun the process of Accession to the European Union, it has signed and ratified numerous international regulations, conventions and recommendations related to persons with mental disabilities, among which the Council of Europe Additional Protocol to the Convention on Human Rights and Biomedicine on Biomedical Research and the UN Convention on the Rights of Persons with Disabilities [7, 19]. Therefore, the country is obligated to move away from its present-day regime of laws that promote and empower state and other people to make choices on behalf of these persons suffering from severe mental disorders. Given that current regime appears to be based on the paternalistic assumption that these persons are not able to exercise their right to choose on the equal basis with others, Montenegro should make every effort to set out a comprehensive framework of measures to address the existing discrimination wrapped up in “best-interest” policies, and to promote equal opportunities for persons with mental disabilities to participate in healthcare and research, as well as in society in general.

It would be well advised that the government overrule the 2013 Law on Protection and Exercise of the Rights of the Mentally Ill [2], and return to the previous state of affairs and the 2005 version of this law which was in accordance with the Council of Europe Additional Protocol to the Convention on Human Rights and Biomedicine on Biomedical Research, as pertains to the article on research on those participants who cannot consent for themselves due to their mental illness [7, 10].

Additionally, since the Additional protocol to the Convention on Human Rights and Biomedicine on Biomedical Research suggests that for research involving incapacitated participants, but which offers no prospect of direct benefit, the risks and burdens should be minimal [7], I also recommend discreetly lifting this very low “risk ceiling” up to a minor increase above minimal risk. Although the concept of minimal risk is an acceptable standard, it could be limiting and prevent important research that might obtain valuable knowledge [32]. The research conducted on these persons needs to be directed at contributing to the scientific understanding of the individual’s condition and obtaining results that will benefit either the participants or other persons in the same category or having the same condition that renders them unable to consent [7]. The recommendation for a slight increase of research risks for participants is based on the CIOMS 2016 revision of International Ethical Guidelines for Health-related Research Involving Humans [8], which acknowledges the concept of minimal risk as an acceptable standard, but also recognizes that procedures involving a minor increase above minimal risk would not pose a significant threat to the participant’s wellbeing. In accordance with the CIOMS guidelines, it should be allowed for research ethics committees to permit this level of risks for studies of compelling social value, which cannot be conducted with persons who can provide informed consent [8].

There is, generally speaking, no precise formula for accessing an appropriate risk-benefit ratio, so understanding the context in which a study is conducted is important for this evaluation. Prior to adjusting its legislative framework and adopting a new set of bills, the Montenegrin government should also carry out consultations with the individuals and communities to be involved in research, in order to determine their values and preferences and to understand what they consider to be favorable benefits and acceptable risks [8]. Engaging with individuals and communities strengthens their role as stakeholders in research, and could help identify the needs of a particular group of participants or identify additional risks that might not have been previously recognized or appreciated by the investigators or the IRB. These individuals and communities may even suggest potential solutions to minimizing risks, identifying additional research questions, or enhancing benefits both to the participants and to the group of participants for which the research is designed [33].

Montenegrin legislation on restriction of legal capacity for the mentally disabled persons, as set out in the 2015 Civil Procedures Act [20], has largely been outdated and is not in line with the international legal framework. Therefore, the government should take steps to correct the existing restrictive measures and establish legal solutions that promote supported decision-making, so that persons with disabilities due to their cognitive impairments can rightly exercise their legal capacity. I recommend that Montenegro abolishes current regime and move away from substitute decision-making through guardianship, and instead to support persons with mental disabilities to exercise their right to make choices for themselves. There must, of course, still be circumstances in which the right to make decisions may be denied. Under those circumstances, when a person is deemed to lack capacity to make particular decisions, a Court should appoint a decision-making trustee, while the will and preferences of the individual in question are taken into account to the largest extent possible.

Although different mental disorders may compromise an individual’s decision-making ability, it should not be assumed that a mentally ill individual lacks the capacity to appreciate the information and implications of participation in research or the capacity to consent to research. Indeed, there is evidence that people with schizophrenia and related psychoses commonly retain decision making capacity for research, despite lacking decision making capacity for treatment [34]. Evidence also suggests that other severely mentally ill patients can maintain substantial decisional capacity and be able to make choices that appear objectively reasonable [35]. Findings further propose that rather than assuming the loss of decision-making ability for individuals who suffer from severe mental disorders, emphasis should be on adequate assessment of their capacity and its remediation in the informed consent process [36]. In many cases, impairment of their decision-making capacity can be compensated for by applying more intensive educational interventions and providing them with additional opportunities to learn the necessary data. Implementation of these techniques could be a part of the informed consent process and augment the efforts to improve their ability to provide consent [35, 36].

The need for individualized capacity assessment of prospective participants is additionally necessitated by the notion that it would be equally wrong to show lack of respect for an autonomous agent, as it would be not to provide additional safeguards for a non-autonomous one [37]. The issue of when to assess capacity is also important in research with the mentally ill. A person should be assumed to have capacity unless proven otherwise, yet there must be a protocol that addresses the issue of determining when a potential participant should be assessed for consent capacity or how the assessment should be performed. I recommend employing the fairly clear guidance as offered through The United Kingdom (UK) Mental Capacity Act of 2005. This Act recommends that the reasons for questioning a person’s capacity to make decisions at a particular time [38] include:

- The person’s behavior or circumstances raise doubt as to whether they have the capacity to make a decision;
- A family member or a healthcare worker have raised concerns about a person’s capacity;
- The person is previously or currently diagnosed with a condition that is known to cause impairments to their decision-making;
- It has already been established that a person lacks capacity to make decisions.

Another important issue that must be defined in the context of psychiatric research is the definition of adults who are unable to make decisions for themselves or the minimum threshold for decision-making capacity. Again, I recommend utilizing guidelines from The UK Mental Capacity Act [38]. The legal framework, as set out under this Act, is designed to protect those who lack decision-making capacity while also maximizing their ability to make decisions or to participate in decision-making as far as possible. It states that a person is unable to make a decision for themselves if there is an impairment of, or disturbance in the functioning of a person’s mind, or brain, and if that impairment or disturbance is sufficient to render them unable:

- To understand the information relevant to the decision;
- To retain that information;
- To use or weigh that information as part of the process of making the decision; or
- To communicate his decision (whether by talking, using sign language or any other means).

In accordance with the same Act, I recommend that informed consent should be regarded as an ongoing process, rather than one-time event. This further implies that even if a person is able to retain the information relevant to a decision only for a short period of time, they are to be regarded as able to understand the information and make relevant decisions. This is particularly important for participants whose capacity is expected to deteriorate or fluctuate, as would be the case with mental disorders like Alzheimer’s disease or schizophrenia. It is suggested that their capacity to provide consent should be re-evaluated at regular intervals during the study and a process for re-consent established [39]. In that way, the principle of respect for persons and their autonomy is reinforced; participants who were previously impaired but who regained their capacity are asked for their personal consent [32].

In compliance with the UN CRPD that the notion of ‘incapacity’ should be unlinked from the notion of mental disability, supported decision making should always be preferred to the substituted decision-making regime [23]. However, it is recognized that there are circumstances in which a person will lack capacity to make a certain decision, and guidelines need to be in place to provide for the use of a LAR to provide permission for research as an alternative to an incapacitated individual’s informed consent. Montenegro should adopt regulations to define and clarify when and who may be appointed as a LAR in the research setting. It should also be noted that the preferred approach to LAR’s making research decisions should be based on their knowledge of prospective participant’s previously stated preferences, objections, values and beliefs [7, 8, 39]. The state should also create means for its citizens to make advanced directives and appoint a person of trust who will act as their future representative, while they still have the necessary legal capacity and the right to state their preference regarding a potential future nomination of legal guardianship [23]. When applicable, if participants have made advanced directives for participation in research while fully capable of giving informed consent, those directives should be respected [8]. If a person left an advanced directive regarding preferred clinical treatments and research, these can also be used to provide LAR with information relevant to individual’s wishes regarding research participation [39]. Finally, under the circumstances in which it is necessary to seek permission from a LAR, the autonomy of the incapacitated participants must be honored by seeking their assent for participation, after providing them with adequate information about the research that is tailored to their capacity to understand. An incapacitated participant’s objection to participate in research, or their subsequent desire to withdraw from it should be respected [7, 8, 39].

## Conclusions

Obtaining consent to participation in psychiatrics research is a complex ethical issue, especially when engaging research participants that are unable to consent for themselves due to the nature of mental disorders they are suffering from. Despite numerous international policies and recommendations on how to practically face this challenge, Montenegro chose not to tackle this issue, by preventing it from ever happening through the lawful ban on research on decisionally-impaired mentally ill subjects. The perhaps well-intended need to protect the group of mentally ill individuals from the potential burdens of research that leads to over-protection, as is the case in Montenegro, may paradoxically allow for their exclusion from the benefits of research that other members of society enjoy. Scientific and clinical development must not be precluded by such overly-restrictive, discriminatory and unjust practices. Rather, regulations should be in place to ensure that persons who are unable to provide consent are not routinely excluded from participation in clinical research.

Creating a normative framework in accordance with established international guidelines that will enable those who suffer from severe mental disorders to participate in research is an imperative, if there is ever a potential that they themselves or those suffering from similar conditions are to benefit from research. These patient-subjects must be appropriately included unless there is a clear and compelling rationale and justification that inclusion is inappropriate with respect to the health of the participants or the purpose of the research. Appropriate safeguards, as suggested in this paper, should be in place to ensure that they are respected in research, the anticipated benefits maximized, risks minimized, their autonomy recognized and extended.

## Data Availability

All data generated or analyzed during this study are included in this published article.

## Abbreviations

CIOMS: Council for International Organizations of Medical Sciences
CRPD: The Convention on the Rights of Persons with Disabilities
EHCI: European Health Consumer Index
IRB: Institutional Review Board
LAR: Legally authorized representative
NIH: National Institutes of Health
UN: United Nations
WHO: World Health Organization
